# Psychophysiological and Neurophysiological Correlates of Dropping Objects from Hands in Carpal Tunnel Syndrome

**DOI:** 10.3390/brainsci13111576

**Published:** 2023-11-10

**Authors:** Gianluca Isoardo, Eugenia Rota, Stefano Ciullo, Paolo Titolo, Enrico Matteoni, Ilaria Stura, Andrea Calvo, Elena Fontana, Bruno Battiston, Giuseppe Migliaretti, Rita B. Ardito, Mauro Adenzato

**Affiliations:** 1 Department of Neurosciences & Mental Health, Hospital “Città della Salute e della Scienza di Torino”, 10126 Turin, Italy; gianluca.isoardo@gmail.com; 2 Neurology Unit, San Giacomo Hospital, Novi Ligure, ASL Alessandria, 15121 Alessandria, Italy; eugenia.rota.md@gmail.com; 3 Department of Psychology, University of Turin, 10124 Turin, Italy; stefanociullo@gmail.com (S.C.); elena.fo@live.it (E.F.); rita.ardito@unito.it (R.B.A.); 4 UOD Reconstructive Microsurgery, Department of Orthopedics & Traumatology, Hospital “Città della Salute e della Scienza di Torino”, 10126 Turin, Italy; titolopaolo@gmail.com (P.T.); bbattiston@cittadellasalute.to.it (B.B.); 5 ‘Rita Levi Montalcini’ Department of Neuroscience, University of Turin, 10126 Turin, Italy; enricomatteoni12@gmail.com (E.M.); ilariastura@gmail.com (I.S.); andrea.calvo@unito.it (A.C.); 6 1st Neurology Unit, Department of Neurosciences & Mental Health, Hospital “Città della Salute e della Scienza di Torino”, 10126 Turin, Italy; 7 Department of Public Health and Pediatric Sciences, University of Torino, 10126 Torino, Italy; giuseppe.migliaretti@unito.it

**Keywords:** carpal tunnel syndrome, neuropathic pain, quantitative sensory testing, cutaneous silent period, cutaneomuscular reflexes

## Abstract

Background: Dropping objects from hands (DOH) is a common symptom of carpal tunnel syndrome (CTS). We evaluated the clinical, neurophysiological, and psychophysiological features of 120 CTS patients to elucidate the DOH pathophysiology. Forty-nine healthy controls were included. Methods: In the patients, the Boston Carpal Tunnel Questionnaire (BCTQ), the Douleur Neuropathique 4 questions (DN4), and a numeric rating scale for pain (NRS) were evaluated. In patients and controls, we evaluated bilateral median and ulnar motor and sensory nerve conduction studies, cutaneous silent period and cutaneomuscular reflexes (CMR) of the abductor pollicis brevis, cold-detection threshold (CDT) and heat-pain detection threshold (HPT) at the index, little finger, and dorsum of the hand, and vibratory detection threshold at the index and little finger by quantitative sensory testing. Results: CTS with DOH had higher BCTQ, DN4 and NRS, lower median sensory action potential, longer CMR duration, lower CDT and higher HPT at all tested sites than controls and CTS without DOH. Predictive features for DOH were abnormal CDT and HPT at the right index and dorsum (OR: 3.88, *p*: 0.03) or at the little finger (OR: 3.27, *p*: 0.04) and a DN4 higher than 4 (OR: 2.16, *p* < 0.0001). Conclusions: Thermal hypoesthesia in median and extra-median innervated territories and neuropathic pain are predictive of DOH in CTS.

## 1. Introduction

Carpal tunnel syndrome (CTS) is the most common entrapment neuropathy [1,2]. The most common symptoms include nocturnal paresthesia, numbness, and pain in the fingers and hand. Weakness of the median-innervated thenar muscles develops at a later stage and is associated with increased severity of CTS [1,2]. Dropping objects is reported by about 50% of patients with CTS and correlates with the severity of CTS [3]. Impairment of dexterity when handling objects has been described in patients with CTS [2,4]. Patients with CTS use more force than controls in both grasping and pinching objects [5,6], and they fail to adjust their effort during dynamic pinching tasks [6]. Force effort and adjustment during the holding task correlated with the degree of median sensory action potential (SAP) amplitude reduction [6], suggesting a critical role of impaired sensory afferent input from median Aβ-fibers during motor control in CTS. However, a bilateral deficit in fine motor control when grasping and pinching has been described in CTS, regardless of the severity of the abnormalities in nerve conduction studies [7]. Furthermore, previous studies [8] reported no correlation between subjective hand weakness and clumsiness, assessed by the Boston Carpal Tunnel Questionnaire (BCTQ) and both sensory nerve conduction studies and vibratory threshold by quantitative sensory testing (QST). In contrast, these motor symptoms were related to pain and warm detection threshold [8]. In other studies, no correlation was found between nerve conduction studies and both the BCTQ Functional Status Scale (FSS) and the difficulty manipulating small objects subscore of the BCTQ Symptom Severity Score (SSS) [9]. These observations suggest that in CTS, (i) the correlation between Aβ-fibers impairment and motor performance is controversial; (ii) small sensory fiber impairment is likely to affect manual dexterity. Both proprioceptive input from muscle spindles and cutaneous sensory afferents play a crucial role in modulating motor performance at the spinal and supraspinal levels [10,11].

Exteroceptive suppression of electromyographic activity can be induced by electrical or mechanical stimulation of the fingers [12,13,14,15]. Low-intensity electrical stimulation of the finger can elicit a complex pattern of inhibition and excitation of voluntary electromyographic activity known as Cutaneomuscular Reflexes (CMRs) [10,12,13,14,15]. In contrast, high-intensity electrical stimulation elicits a transient suppression of EMG activity known as the Cutaneous Silent Period (CSP) [12,13,15,16,17]. CMRs are triggered by the stimulation of Aβ-fibers [10,12,13,14,15], while the afferent input to trigger CSP is mediated mainly by Aδ-fibers and to a lesser degree by Aβ-fibers [12,13,17]. The CSP has been considered a nociceptive reflex that protects the hand from noxious stimuli by inhibiting grasping and pinching [12,13,15]. CMRs are considered part of the regulatory mechanism for tuning hand movements, including grip [10]. The late inhibitory component of CMRs in the abductor pollicis brevis [12,14,15] is likely supplied by a transcortical circuit and may represent the late part of CSP [15]. The duration of CSP, elicited by high-intensity stimulation of the index finger, is increased in the abductor pollicis brevis (APB) in patients with mild to moderate CTS, while it decreases to a normal value or is even shortened in severe CTS [15,18,19]. In a previous study [16], we found that in CTS patients, the duration of the CSP was strongly and significantly correlated with the latency to offset of CSP. Since the late part of the CSP seems to be formed by the late inhibitory component of CMRs, the prolongation of the CSP in CTS could be due to an increased duration of this late inhibitory component of CMRs. Since the CMRs are involved in fine-tuning hand movements, abnormalities in the late inhibitory component of the CMRs could correlate with impaired manual dexterity in CTS.

QST is a psychophysiological evaluation that allows the detection of abnormalities of various somatic sensory modalities such as cold, warm or heat-pain, vibration or touch/pressure detection [16,20,21,22]. QST is considered particularly helpful in the diagnosis of neuropathic pain [16,21,22], and standardized QST algorithms have been developed [20].

The aim of the present study is to determine which clinical, neurophysiological (including CMRs and CSP), and psychophysiological (QST) abnormalities predict dropping objects from the hands in patients with CTS. In addition to nerve conduction studies and BCTQ, we prospectively performed QST, CSP and CMRs assessment in a cohort of patients with CTS and age- and sex-matched healthy controls. To our knowledge, no previous studies have evaluated QST performed in median-innervated and extra-median territories of the hand, CSP and CMRs to further elucidate the pathophysiology of dropping object from hands in a large cohort of patients with CTS.

## 2. Material and Methods

### 2.1. Patients

We enrolled patients with CTS who were referred to our neurophysiology laboratories or to the microsurgery outward. The diagnosis of CTS was based on the clinical and neurophysiological criteria of the American Academy of Neurology [23] and the American Association of Electrodiagnostic Medicine [24]. Forty-nine healthy control subjects were recruited from the investigators, their family members and the hospital’s nursing and administrative staff. All voluntarily underwent a complete medical history collection, neurological examination and nerve conduction studies evaluation to rule out CTS and other neurological disorders. With the exception of clinical and laboratory evidence of CTS in the healthy controls, the same exclusion criteria applied to the patients and healthy controls: age under 18; inability to complete the QST examination with sufficient accuracy [16]; family history of inherited neurological disease; history or clinical or laboratory evidence of cervical radiculopathy, myelopathy, polyneuropathy, multiple mononeuropathy affecting nerve trunks other than the median nerve at the wrist, or other neurological diseases. The BCTQ [4] and the Douleur Neuropathique 4 questions (DN4) [25] were evaluated in all patients. Self-reported pain intensity was graded on an 11-point numerical rating scale (NRS), with scores ranging from 0 (no pain) to 10 (worst possible pain) [26]. All patients underwent a full clinical evaluation, including Medical Research Council (MRC) scale scores for APB and abductor digiti minimi, pinprick, touch, and assessment of position sense in both upper limbs. Allodynia during brushing was evaluated at the sites of pain. To evaluate manual dexterity, the nine-hole peg test (9HPT) was performed [27]. To assess manual dexterity, patients and controls also underwent a modified 9HPT (m9HPT) using a checkers game board with 2 mm pieces. In this m9HPT, all 24 pieces had to be picked from a box and inserted in little holes on the game board as quickly as possible. In addition to assessing manual dexterity, complaint and the frequency of dropping objects from the hands were also assessed by asking the patients directly, as in the study by Pazzaglia et al. [3]. For the purpose of this study, patients were divided into those who complained about dropping objects from their hands (Group 1) and those who did not (Group 2). Group 1 was further divided according to the size of the objects more frequently dropped from hands, as follows: dropping small objects such as a pencil or a spoon (Group 1-s); dropping large objects such as a frying pan or a grocery bag (Group 1-l); dropping both large and small objects (Group 1-s/l).

### 2.2. Neurophysiological Assessment

Patients underwent bilateral motor nerve conduction studies of the median and ulnar nerves and antidromic sensory NCS of the median and ulnar nerves as previously described [16,25], using commercially available electrodiagnostic equipment (Viking Quest, Carefusion, Middleton, WI, USA). Comparison of antidromic median and ulnar sensory latency at fourth digit was performed as described [25]. The presence of concurrent ulnar neuropathy at the elbow and cervical radiculopathy was defined as previously described [25].

CSP and CMR of the APB were recorded with surface electrodes in a bipolar belly-tendon montage, after the index finger was electrically stimulated. Patients and control subjects performed an isometric contraction at maximum force against a resistance and received audio feedback to maintain constant the contraction strength. Although maximal contraction tends to reduce the duration of CSP and CMRs [12,13], we decided to perform the assessment of both exteroceptive electromyographic activity suppressions at this force level to (i) make the results comparable to those previously reported by the same group [16] and to (ii) reduce the late excitatory component of CMR, as shown in APB [14]. Stimulation was delivered through ring electrodes, with the cathode placed at the proximal interphalangeal joint of the second digit. CSP was obtained after stimulation at an intensity 8 times the perceptual threshold for an electric shock, while CMR was at an intensity 2 times the perceptual threshold. This threshold was determined separately for each hand by slowly increasing the intensity of stimulation delivered at 1 Hz until the patients perceived a sensation of a non-painful electric shock. Electromyographic activity was rectified and averaged over 8 trials in each hand for CSP. For CMR, the electromyographic signal was rectified, and each of the 10 traces that showed suppression of activity lasting more than 10 ms in duration was included for analysis. To avoid habituation, each trial was performed at least 60 s after the previous trial. Onset and offset of CSP and CMR were defined by visual inspection as the beginning of an abrupt decrease and recovery of electromyographic activity, as previously described [16].

### 2.3. Psychophysiological Evaluation—Quantitative Sensory Testing

QST was performed to determine the thresholds for the perception of cold (CDT), heat-induced pain (HPT), and vibration (VDT) [16,25]. CDT, HPT, and VDT were measured on the palmar surface of the index and little finger. In addition, CDT and HPT were also measured on the dorsum of the hand, using a commercially available thermal stimulation device (Medoc TSA II, Durham, NC, USA). HPT was assessed using the method of limits [16,21]. Stimulation began at 32 °C and increased by 1 °C per second until the participant perceived a change from heat sensation to pain or the probe temperature reached 50 °C. Five trials at each site were averaged. CDT was evaluated using a staircase method with null stimulations [16,21]. Briefly, three ranges of steps of cooling are presented, beginning with a gross 3 °C decrease in temperature. Stimulation began at 32 °C, and the participant was asked to indicate whether or not they perceived the cooling step. VDT was assessed using the method of levels with null stimulations [21]. The stimulation started at 0 µm. The QST was considered insufficiently accurate if participants could not identify at least two of five null stimuli during CDT and/or VDT assessment. The CDT and HPT results were log-transformed, and the VDT results were ln-transformed [16,20,21]. Then, the patients’ z-scores were calculated for each modality and site [16,20,21]. 

Hypoesthesia for cold and vibration was defined when the z-scores for CDT were lower than −2.58 and when those for VDT were higher than 2.58. Hypoesthesia for heat was defined when the z-score for HPT was higher than 1.64. Allodynia for heat pain was defined when the z-score was lower than −1.64.

### 2.4. Statistical Analysis

All descriptive statistics for continuous variables were expressed as means ± standard deviation (SD), while categorical variables were reported as frequencies and percentages. Differences between groups were tested using Fisher’s exact test for categorical variables and the Mann-Whitney or Wilcoxon test for continuous variables. Correlations were calculated using Pearson’s product-moment correlation and reported with r coefficients. Results were corrected for multiple comparisons. Analysis of data from patients with bilateral CTS may overestimate statistical significance if only the hands are compared [16,21]; so, statistical analysis was performed for both hands and patients [16,21]. Logistic regression models were performed to evaluate which clinical, neurophysiological, or QST parameters predicted dropping objects from the hands, both for the totality of patients and for subdivision according to the dimension of the dropping objects. Results were expressed as Odds Ratio (OR) with 95% Confidence Intervals (95% CI). For each test, the *p*-value is reported, with 0.05 as the significance threshold (I species error α = 0.05). All analyses were performed using SAS^®^ Statistics Software version 9.4.

## 3. Results

### 3.1. Demographic and Clinical Features

One hundred and twenty patients with CTS and forty-nine healthy controls were included in the study. Demographic and clinical data are summarized in Table 1. There were no differences in age and sex between patients and controls. CTS affected the right hand in 32 patients, the left hand in 13 and both hands in 75, so that a total of 195 hands with CTS were included for analysis. 

Seventy-five patients (62.5%) reported dropping objects from their hands (Group 1), while forty-five did not (Group 2). No significant difference in the frequency of dropping objects from the hands was found between patients with bilateral (47 of 75), right (20 of 32), or left CTS (8 of 13), and age and sex did not differ between Group 1 and Group 2. Compared with Group 2, Group 1 had a higher DN4, NRS, BCTQ total score, BCTQ symptom severity score (SSS), BCTQ Functional Severity Score (FSS), and all BCTQ subscores, except those for the presence of tingling sensation and frequency of nighttime wake up due to tingling or numbness (Table 1). The time to perform the m9HPT bilaterally was higher in Group 1 than both Group 2 and healthy controls. The time to perform the 9HPT was higher in Group 1 than in the healthy control subjects. The MRC score of bilateral APB was lower in Group 1 than in the healthy controls. Group 1-s showed no difference from Group 2 in BCTQ subscore for daytime pain and NRS, while Group 1-l showed a difference in BCTQ subscore for difficulty in manipulating small objects and m9HPT bilaterally.

### 3.2. Nerve conduction Studies

Nerve conduction studies are summarized by patient in Table 2 and by hand in Supplementary Table S1. Bilateral median motor (MCV), median sensory conduction velocities (SCV) and both median and ulnar amplitude of SAP were significantly lower in Group 1 and Group 2 than in controls. The distal motor latency (DML) of the median compound muscle action potential (CMAP) was significantly longer in both Group 1 and Group 2 than in healthy controls. The amplitude of the right median CMAP was lower in Group 1, Group 1-l, and Group 1-s/l than in healthy control subjects. Right median SCV and SAP amplitudes were lower in Group 1 and Group 1s/l than Group 2. When compared by hand, hands with CTS had lower median SCV, MCV, median SAP, and CMAP amplitudes, ulnar SAP amplitude, and higher median DML than hands of patients without CTS (no-CTS) and hands of healthy controls. No-CTS hands had lower median MCV, SCV, median and ulnar SAP amplitude and higher median DML than the hands of healthy controls (Supplementary Table S1).

### 3.3. Cutaneomuscular Reflexes and Cutaneous Silent Period

The results of the CMR and CSP are summarized by patient in Table 3 and by hand in Supplementary Table S1. Right CMR onset latency was significantly shorter in Group 2 than in the healthy controls. Compared with healthy controls, CMR duration was higher in the right hand in Group 1 and bilaterally in Group 1-s. In the right hand, Group 1-s had higher CMR latency and duration than Group 2. Hands with CTS had both CSP and CMR duration higher than healthy controls’ hands (Supplementary Table S1). The current intensities to induce both CMR and CSP were higher on both the hands with CTS and the no-CTS hands than the healthy controls’ hands (Supplementary Table S1).

### 3.4. Quantitative Sensory Testing

QST results are summarized in Table 4, Supplementary Tables S2 and S3. Group 1 had lower log-transformed CDTs and higher ln-transformed VDTs than the healthy controls at all sites evaluated. In contrast, no difference was found between Group 2 and the healthy controls in all QST parameters except ln-transformed VDT at the index bilaterally. The CDT z-score of Group 1 and Group 1-s was lower at the right little finger than that of Group 2. The HPT z-score at the right dorsum and index was higher in Group 1 and Group 1-s/l than in Group 2, and at the little finger in Group 1-s/l than Group 2. When comparisons were performed by hands, CTS hands had lower log-transformed CDTs and higher ln-transformed VDTs than healthy controls’ hands at index and little finger (Supplementary Table S2). Index ln-transformed VDT was higher in the no-CTS hands than in the healthy controls.

### 3.5. Correlation between Boston Carpal Tunnel Questionnaire and Other Clinical Evaluations

The right APB MRC score correlated with BCTQ-SSS (r: −0.30, *p*: 0.001), BCTQ-FSS (r: −0.34, *p*: 0.0004), BCTQ overall (r: −0.36, *p*: 0.0001) and DN4 score (r: −0.4, *p* < 0.0001), while the left APB MRC score correlated with BCTQ FSS (r: −0.42, *p* > 0.0001) and BCTQ overall (r: −0.36, *p*: 0.0001). Time to perform the m9HPT with the right hand correlated with BCTQ SSS subscore evaluating difficulty in grasping and manipulating small objects (r: 0.40, *p*: 0.0007), BCTQ overall (r: 0.36, *p*: 0.002), while the time to perform m9HPT bilaterally correlated with BCTQ FSS (right r: 0.41, *p*: 0.0005; left r: 0.41, *p*:0.001), BCTQ FSS subscores for buttoning clothes (right r: 0.39, *p*: 0.001; left r: 0.41, *p*: 0.001), handing a book while reading (right r: 0.36, *p*: 0.002; left r: 0.476, *p* < 0.0001), opening of jars (right r: 0.34, *p*: 0.004; left r: 0.32, *p*: 0.009), household chores (right r: 0.38, *p*: 0.001; left r: 0.34, *p*: 0.005), bathing and dressing (right r: 0.39, *p*: 0.001; left: r: 0.33, *p*: 0.008). In contrast, the time to perform 9HPT bilaterally correlated only with the BCTQ-FSS subscore for buttoning clothes (right r: 0.44, *p*: 0.002; left r: 0.42, *p*: 0.004).

### 3.6. Correlations between Neurophysiological and Psychophysiological Parameters

In hands with CTS, median SAP amplitude correlated with CMR duration (r: −0.24, *p*: 0.001), ulnar SAP amplitude (r: 0.61, *p* < 0.0001), median CMAP amplitude (r: 0.5, *p* < 0.0001) and DML (r: 0.49, *p* < 0.0001), VDT z-score at the index (r: −0.25, *p*: 0.02) and at the little finger (r: −0.33, *p*: 0.0003). The median SCV correlated with the median CMAP amplitude (r: 0.31, *p* < 0.0001) and DML (r: −0.77, *p* < 0.0001), VDT z-scores at the index (r: −0.41, *p*: 0.0002) and little finger (r: −0.38, *p*:0.0007). In no-CTS hands, median SAP amplitude correlated with offset latency of CMR (r: −0.62, *p*: 0.01) and CSP (r: −0.58, *p*: 0.001), ulnar SAP amplitude (r: 0.75, *p* < 0.0001) and VDT z-score at the index (r: −0.46, *p*: 0.02). The median SCV correlated with the ulnar SCV (r: 0.48, *p*: 0.002). In healthy control hands, median SAP amplitude correlated with median SCV (r: 0.43, *p*: 0.0009), ulnar SAP amplitude (r: 0.88, *p* < 0.0001), median CMAP amplitude (r: 0.5, *p*: 0.0001). The median SCV correlated with the offset latency of the CMR (r: −0.49, *p*: 0.01).

In CTS hands, CMR duration correlated with onset latency (r: −0.38, *p* < 0.0001), offset latency (r: 0.42 *p* < 0.0001), intensity of stimulation (r: 0.45, *p* < 0.0001), and CSP duration (r: 0.57, *p* < 0.0001). In no-CTS and healthy control hands, CMR duration correlated only with onset latency (r: −0.58, *p*: 0.02 and r: −0.61, *p*: 0.0009, respectively). In hands with CTS, no-CTS and healthy control hands, the CSP duration correlated with onset latency (respectively r: −0.41, *p* < 0.0001; r: −0.49, *p*: 0.01 and r: −0.32, *p*: 0.01) and offset latency (respectively r: 0.85, *p* < 0.0001; r: 0.68, *p*: 0.0001 and r: 0.85, *p*< 0.0001).

### 3.7. Correlation between Clinical Evaluations and Neurophysiological Parameters

BCTQ SSS subscore evaluating difficulty in grasping and manipulating small objects correlated with bilateral median SAP amplitude (right r: −0.26, *p*: 0.004; left r: −0.24, *p*: 0.04), right CMR duration (r: 0.23, *p*: 0.002), right ulnar SAP amplitude (r: −0.2, *p*: 0.02) and bilateral ulnar SCV (right r: −0.21, *p*: 0.02; left r: −0.28, *p*: 0.02). BCTQ FSS subscores for bathing and dressing correlated with bilateral CMR duration (right r: 0.27, *p*: 0.004 and left: r: 0.27, *p*: 0.03). In hands with CTS, the time to perform m9HPT correlated with CMR duration (r: 0.31, *p*: 0.002), CMR offset latency (r: 0.25, *p*: 0.02), the CSP offset latency (r: 0.29, *p*: 0.003), the median SAP amplitude (r: −0.50, *p*: 0.009), and the ulnar SAP amplitude (r: −0.45, *p*: 0.02).

### 3.8. Correlation between Clinical Evaluations and Psychophysiological Parameters

The right dorsum CDT z-score correlated inversely with the BCTQ FSS subscores for bathing and dressing (r: −0.31, *p*: 0.02) and opening of jars (r: −0.30, *p*: 0.02); the CDT z-score of the right index finger correlated inversely with the BCTQ SSS subscore evaluating difficulty in grasping and manipulating small objects (r: −0.32, *p*: 0.02), BCTQ FSS subscores for bathing and dressing (r: −0.44, *p*: 0.0003) and buttoning clothes (r: −0.28, *p*: 0.02). The CDT z-score of the right little finger correlated with overall BCTQ (r: −0.36, *p*: 0.005), BCTQ-FSS (r: −0.41, *p*: 0.001), BCTQ FSS subscores for buttoning clothes (r: −0.33, *p*: 0.01), holding a book while reading (r: −0.44, *p*: 0.0004), opening jars (r: −0.34, *p*: 0.007), doing household chores (r: −0.31, *p*: 0.01), bathing and dressing (r: −0.46, *p*: 0.0003). The left index CDT z-score correlated with the BCTQ FSS subscore for bathing and dressing (r: −0.35, *p*: 0.007). In hands with CTS, the time to perform the m9HPT correlated with the dorsum and index CDT z-scores (respectively, r: −0.45, *p* < 0.0001, r: −0.48, *p* < 0.0001).

### 3.9. Predictive Features for Dropping Objects from Hands

Predictive features for dropping large objects from hands are the detection of both CDT z-score lower than 2.58 and HPT z-score higher than 1.64 at both right index and dorsum (OR: 3.88, 95% CI 1.12–13.4, *p*: 0.03) or both the right index and little finger (OR: 3.27, 95% CI 1.02–10.4, *p*: 0.04). A DN4 score higher than 4 is also predictive of dropping objects from hands (OR: 2.16, 95% CI 1.57–2.97, *p* < 0.0001; small objects OR: 2.47, 95% CI 1.53–3.93, *p*: 0.0002; large objects OR: 1.82, 95% CI 1.3–2.56, *p*: 0.0004; large and small objects OR: 2.25, 95% CI 1.45–3.52, *p*: 0.0003).

## 4. Discussion

In this study, we have shown that CTS patients who complain of dropping objects from their hands have distinct clinical features and neurophysiological and QST abnormalities compared to CTS patients who do not complain of dropping objects. Compared to patients in Group 2, patients in Group 1 had higher overall BCTQ score, BCTQ-SSS, BCTQ-FSS, DN4, and NRS, indicating higher symptom severity and impaired functional status of CTS, as well as higher neuropathic pain and pain intensity.

These results are consistent with previous reports [3,8]. Tamburin et al. [8] defined hand clumsiness based on the BCTQ-SSS subscore for difficulty in manipulating small objects. In their study, hand clumsiness was related to pain, sensory symptoms, and impairment in warm detection threshold by QST in the median nerve innervation territory, but they found no association with CDT. In our study, the presence of neuropathic pain, defined by DN4, increased the likelihood of dropping objects from the hands by 2-fold, and abnormal CDT and HPT indicative of sensory loss in median and non-median innervated territories increased the likelihood of dropping large objects from the hands by more than 3-fold. The discrepancy between our study and that of Tamburin et al. [8] on the relationship between CDT and motor control may be due to differences in QST methodology and statistical analysis of QST results. Tamburin et al. [8] did not log-transform the QST results and did not evaluate z-scores, in contrast to the suggestions of the German Neuropathic Pain Research Network [20]. Furthermore, they evaluated CDT with a reaction time-dependent method of limits, whereas in our study, CDT and VDT were evaluated with reaction time-independent staircase and levels’ methods, respectively [21].

We found a relationship between the right index CDT and BCTQ-SSS for difficulty in manipulating small objects. Patients in Group 1, but not those in Group 2, had lower log-transformed CDT than healthy controls at all sites examined, and right little finger CDT in CTS correlated with overall BCTQ, overall BCTQ-FSS, and BCTQ-SSS subscores for grasping small and large objects. These observations further support the role of cutaneous afferents and integration of sensory inputs from median and non-median innervated territories in grasping impairment in CTS [5,6]. Extra-median spread of symptoms and QST abnormalities have been described in patients with CTS without evidence of ulnar or radial nerve damage [28,29]. Furthermore, both painful and non-painful unilateral nerve or radicular damage are associated with contralateral sensory loss, including abnormal CDT and VDT [30]. In our study, ln-transformed VDT and electric shock detection threshold at index were higher in the hands of patients without CTS than in the hands of healthy controls. In our study, ulnar nerve conduction parameters were within the normal ranges in all patients and control subjects. Nevertheless, ulnar SAP amplitude on both sides was lower in CTS patients than in healthy controls and in both CTS hands and no-CTS hands than healthy controls’ hands, as previously described [31]. This significant, albeit subtle, reduction in ulnar nerve SAP amplitude did not correlate with dropping objects from the hands, in contrast to the QST abnormalities in the extra-median territory. These observations suggest that the extra-median spread of symptoms and QST abnormalities may be related to plastic changes in the central nervous system. Maeda et al. [32] reported that reduced distance of the cortical representation of the median-innervated hand fingers correlated with BCTQ-SSS and subscore for paresthesia, accuracy of sensory discrimination by median-innervated fingers, and pinch-release task performance. The m9HPT in our study is similar to a pinch-release motor task, and the CTS patients had significantly prolonged time to perform the m9HPT than healthy controls.

Grasping can be performed differently by the hand depending on object characteristics, including size. Power and grip precision are controlled by different pathways [33]. Muir and Lemon [34] described a subset of pyramidal tract neurons in the monkey motor cortex that show a selectively increased firing rate during precision grip. Cutaneous afferents stimulation reduces the activity of the low-threshold, slow-twitch motor unit innervating spinal motor neurons and increases the activity of the high-threshold, fast-twitch motor unit innervating them [35]. In our study, CMR duration in the right hand ABP was increased only in patients who complained of dropping small objects. Increased CMR duration in hands with CTS may reflect disruption of the transcortical loops necessary for adaptation of grip force [36] rather than impaired conduction along the Aβ-fiber, as suggested by the lack of association with median SAP amplitude, SCV, and VDT at index. In contrast, CMR duration is related to the BCTQ SSS subscore for manipulating small objects, is prolonged in patients complaining of dropping small objects, and is related to time to perform the m9HPT in hands with CTS. These observations suggest that disruption of the transcortical loop involved in CMR generation in ABP may contribute to impairment of small objects manipulation ability/precision pinch in CTS. It is noteworthy that CSP, which is considered a nociceptive reflex subserved mainly by a spinal circuit [12,13,17], is not associated with object manipulation.

### 4.1. Limitations of the Study

A possible limitation of the study is the sample size, which could reduce the possibility to detect differences in predictive features for dropping objects in patients with bilateral, right, and left hands CTS. Another possible limitation is the lack of QST evaluation at more proximal sites of the arm to better define the extra-median spread of sensory abnormalities in CTS patients with and without dropping objects from hands.

### 4.2. Conclusions

In summary, our results suggest that dropping objects from hands in patients with CTS is related to: (i) impairment of small-fiber conveyed sensation in median and extra-median innervated territories and (ii) presence of neuropathic pain defined by a DN4 score higher than 4. Furthermore, prolonged duration of CMRs in the right abductor pollicis brevis is related to dropping small objects from hands. These observations suggest a possible involvement of cutaneous afferents and adaptive changes in the central nervous system in the dropping of objects from hands in CTS patients.

## Figures and Tables

**Table 1:** Demographic and clinical features of patients and controls.

		Carpal Tunnel Syndrome				Healthy Controls
		Dropping Objects			No Dropping Objects	
	All	Small	Large	Large/Small		
Age	56.1 ± 12.4	57.1 ± 10.4	56.8 ± 13.6	57.5 ± 14.4	57.6 ± 12.5	53.1 ± 12.6
Male/Female	27/93	6/22	4/25	5/12	12/34	15/35
BCTQ						
Total score	45.4 ± 13.9 *	49.8 ± 15.8 *	50.5 ± 11 *	50.5 ±11.0 *	36.5 ± 10.2	
SSS	32.5 ± 8.4 *	31.4 ± 9.9 *	33.2 ± 7.1 **	33.1 ± 8.5 *	23.9 ± 7.2	
Pain at night	2.7 ± 1.4 *	2.9 ± 1.4 **	3.2 ± 1.1 *	2.9 ± 1.6 **	2.1 ± 1.3	
Night wake-up by pain	2.4 ± 1.5 *	2.4 ± 1.4 *	3.3 ± 1.4 **	2.9 ± 1.6 **	1.7 ±1.2	
Daytime pain	2.4 ± 1.2 *	2.3 ± 1.2	3.1 ± 1.1 **	2.7 ± 1.3 *	2 ± 1	
Daytime pain frequency	2.3 ± 1.4 *	2.8 ± 1.5 **	3.2 ± 1.2 *	3 ± 1.4 **	2.2 ± 1.3	
Daytime pain duration	2.3 ± 1.3 *	2.5 ± 1.4 **	2.8 ± 1.3 **	2.5 ± 1.3 **	1.8 ± 1.1	
Numbness	2.8 ± 1.3 *	3.2 ± 1.1 **	3 ± 1.2 **	3 ± 1.2 **	2.3 ± 1.3	
Weakness	2.3 ± 1.1 *	2.8 ± 1.1 *	2.6 ± 0.5 *	2.7 ± 1 *	1.5 ± 0.8	
Tingling	3.3 ± 1.1	3.2 ± 1.2	3.6 ± 0.9	3.3 ± 0.8	3.1 ± 1.1	
Numbness severity	3.3 ± 1.2 *	3.3 ± 1.2	3.7 ± 1.2 **	3.9 ± 0.9 **	2.9 ± 1.2	
Night wake-up by numbness	2.9 ± 1.4	3.2 ± 1.4	3.0 ± 1.4	3.1 ± 1.4	2.4 ± 1.4	
Grasping small objects	2.2 ±1.3 *	2.8 ± 1.3 *	2.1 ± 1.1	2.6 ± 1.4 **	1.7 ± 1.1	
FSS	18.4 ± 6.9 *	18.4 ± 8 **	17.3 ± 6.3 **	20.5 ± 5.7 **	12.6 ± 5.1	
Writing	1.8 ± 1.1 **	2.0 ± 1.2 **	1.9 ± 0.9 **	2.2 ± 1.1 **	1.5 ± 1.0	
Buttoning clothes	1.9 ± 1.2 *	2.2 ± 1.1 *	2.2 ± 1.1 **	2.5 ± 1.1 *	1.5 ± 1.0	
Holding books	1.6 ± 1 *	1.9 ± 1.1 *	1.8 ± 1.2 **	1.9 ± 0.8 **	1.2 ± 0.7	
Gripping phone	1.6 ± 0.9 *	1.9 ± 1.0 **	1.7 ± 1.0 **	2.1 ± 1.0 *	1.3 ± 0.6	
Opening jars	2.9 ± 1.4 *	3.0 ± 1.4 **	3.3 ± 1.5 *	3.7 ± 1.3 *	2.2 ± 1.2	
Household chores	2.1 ± 1.1 *	2.5 ± 1.2 *	2.2 ± 1.0 **	2.7 ± 1.1 *	1.5 ± 0.9	
Carrying grocery bags	2.7 ± 1.5 **	3.0 ± 1.5 **	2.7 ± 1.3	3.4 ± 1.2 **	2.3 ± 1.4	
Bathing and dressing	1.5 ± 0.8 *	1.8 ± 1.1 *	1.5 ± 0.9 **	2.0 ± 0.9 *	1.1 ± 0.4	
DN4	5.9 ± 1.8 *	5.9 ± 1.6 *	5.5 ± 1.9 *	6.6 ± 1.9 *	3.2 ± 1.9	
NRS	6.3 ± 2.4 **	5.5 ± 2.7	6.7 ± 2.2 **	7 ± 2.1 **	4.4 ± 3	
9HPT (s)						
Right	13.8 ± 2.6 ^δ^	14.7 ± 1.2 *^§^	14.7 ± 3.4 ^§^	12.4 ± 1.7	12.9 ±1.6 ^§^	12.2 ± 0.9
Left	14.9 ± 2.9 ^§^	14.5 2 ± 1.5	15.9 ± 3.5 ^§^	13 ± 0.99	14.7 ± 2.5	13.1 ± 1.9
m9HPT (s)						
Right	170.2 ± 70.8 *^δ^	192.8 ± 84.9 *^δ^	188.2 ± 72 ^§^	174.5 ± 64.9 ^§^	144.7 ± 49.7 ^§^	126.2 ± 38.0
Left	169.5 ± 62.4 *^δ^	177.3 ± 54.1 *^δ^	199.6 ± 88.1 ^§^	190.7 ± 62.9 *^δ^	147.8 ± 49.3 ^§^	129.1 ± 39.1
MRC score APB						
Right	4.5 ± 0.7 ^§^	4.5 ± 0.7 ^§^	4.5 ± 0.7 ^§^	4.1 ± 0.7 *^δ^	4.6 ± 0.6	5
Left	4.3 ± 0.9 ^§^	4.2 ± 1.1 ^§^	4.4 ± 0.8 ^§^	4.0 ± 1.2 *^§^	4.6 ± 0.6	5

APB: abductor pollicis brevis; BCTQ: Boston Carpal Tunnel Questionnaire; DN4: Douleur Neuropathique 4 questions; FSS: functional status scale; 9HPT: 9-hole peg test; m9HPT: modified 9-hole peg test: MRC: Medical Research Council Scale; NRS: numerical rating scale; s: seconds; SSS: symptom severity scale; * *p* < 0.0001 vs. patients not complaining of dropping objects; ** *p* < 0.05 vs. patients not complaining of dropping objects; ^δ^ *p* < 0.0001 vs. healthy controls; ^§^ *p* < 0.05 vs. healthy controls.

**Table 2:** Nerve conduction studies in patients and controls.

		Carpal Tunnel Syndrome				Healthy Controls
		Dropping Objects			No Dropping Objects	
	All	Small	Large	Large/Small		
Median NCS						
SCV (m/s)						
Right	41.6 ± 7.9 **^δ^	40.9 ± 9.3 ^δ^	42.5 ± 7.8 ^δ^	39.1 ± 7.3 **^δ^	42.7 ± 7.2 ^δ^	57.9 ± 6.8
Left	45.2 ± 8.4 ^δ^	44.3 ± 8.0 ^δ^	43.4 ± 8.5 ^δ^	43.4 ± 10.0 ^δ^	45.2 ± 8.4 ^δ^	59.1 ± 5.1
SAP-amp (µV)						
Right	19.1 ± 10.0 **^δ^	17.8 ± 15.2 ^δ^	19.4 ± 16.4 ^δ^	14.0 ± 13.1 **^δ^	19.1 ± 10.0 ^δ^	44.4 ± 21.8
Left	25.5 ± 13.5 ^δ^	20.9 ± 17.8 ^δ^	22.6 ± 18.4 ^δ^	19.7 ± 12.4 ^δ^	25.5 ± 13.5 ^δ^	51.2 ± 20.9
MCV (m/s)						
Right	52.8 ± 4.6 ^§^	52.1 ± 3.8 ^§^	53.6 ± 5.4 ^§^	51.8 ± 4.3 ^§^	52.8 ± 4.6 ^§^	56.0 ± 4.8
Left	51.7 ± 9.5 ^δ^	52.8 ± 5.4 ^δ^	53.5 ± 5.2 ^δ^	54.5 ± 3.6 ^§^	51.7 ± 9.4 ^δ^	58.8 ± 4.6
CMAP-amp (mV)						
Right	7.8 ± 3.2 ^§^	8.0 ± 4.3	6.8 ± 2.0 ^§^	7.2 ± 2.6 ^§^	7.8 ± 3.1	8.6 ± 3.4
Left	7.9 ± 3.1	7.8 ± 4.5	6.8 ± 2.6	7.4 ± 3.3	8.0 ± 3.1	9.2 ± 5.4
DML (ms)						
Right	4.5 ± 1.1 ^δ^	4.5 ± 1.7 ^δ^	4.5 ± 1.5 ^δ^	5.4 ± 1.7 ^δ^**	4.5 ± 1.1 ^δ^	3.3 ± 0.4
Left	4.2 ± 1.0 ^δ^**	4.8 ± 1.3 ^δ^	4.5 ± 1.2 ^δ^	4.7 ± 1.4 ^δ^	4.2 ± 1.0 ^δ^	3.3 ± 0.7
Ulnar NCS						
SCV (m/s)						
Right	58.2 ± 5.3	56.5 ± 6.0	56.1 ± 4.3	55.3 ± 4.6	58.2 ± 5.3	58.1 ± 6.2
Left	58.0 ± 7.14	56.7 ± 7.1	56.5 ± 4.4	55.1 ± 3.7	58.0 ± 7.1	58.2 ± 7.0
SAP-amp (µV)						
Right	30.7 ± 14.9 ^δ^	30 ± 15.8 ^δ^	30.7 ± 15.2 ^δ^	28.2 ± 13.1 ^δ^	30.8 ± 14.9 ^δ^	45.5 ± 20.1
Left	33.1 ± 16.3 ^δ^	28.6 ± 16 ^δ^	33.9 ± 18.7 ^δ^	27.6 ± 10.4 ^δ^	33.1 ± 16.3 ^δ^	47.4 ± 23.9

CMAP-amp: compound muscle action potential amplitude; DML: distal motor latency; m: meters; MCV: motor conduction velocity; ms: milliseconds; mV: milliVolt; NCS: nerve conduction studies; s: seconds; SAP-amp: sensory action potential amplitude; SCV: sensory conduction velocity; µV: micronVolt; ** *p* < 0.05 vs. patients not complaining of dropping objects; ^δ^ *p* < 0.0001 vs. healthy controls; ^§^ *p* < 0.05 vs. healthy controls.

**Table 3:** Cutaneomuscular reflex and cutaneous silent period in patients and controls.

		Carpal Tunnel Syndrome				Healthy Controls
		Dropping Objects			No Dropping Objects	
	All	Small	Large	Large/Small		
CMR						
Onset latency (ms)						
Right	91.6 ± 21.4 *	93.6 ± 10 *	104 ± 31.4	90.2 ± 7.8	80.8 ±18.9 ^§^	99.2 ± 18.6
Left	92.9 ± 18.9	96.5 ± 18	90.8 ± 20.8	92 ± 21.7	92.7 ± 19	95.8 ± 13.3
Duration (ms)						
Right	23.7 ± 21.0 ^§^	33.8 ± 18.9 ^δ^*	19.6 ± 17.4	24.5 ± 17.2	22.5 ± 24.6	16.3 ± 15.4
Left	20.3 ± 20.8	22.9 ± 18 ^§^	13.1 ± 17.8	22.8 ± 17.8 ^§^	22.9 ± 25.3	12.4 ±15.7
Offset latency (ms)						
Right	126.9 ± 24.3	129.4 ± 19.5	135.2 ± 38.5	125.2 ± 4.9	121.5 ± 19.4	120 ± 15.3
Left	124.7 ± 15.5	127.7 ± 13.9	116.8 ±14.1	123.1 ± 14.84	130.9 ± 15.2 ^§^	117.9 ± 11.9
Current intensity (mA)						
Right	15.3 ± 7.9 ^δ^	16 ± 6.6 ^δ^	13.6 ± 6.6 ^§^	18.2 ± 10.6	15.7 ± 8.4 ^δ^	9.9 ± 3.7
Left	14.7 ± 8.7 ^§^	16.6 ± 10 ^§^	12.9 ± 7.1	16.7 ± 10 ^§^	14.3 ± 8.6 ^§^	9.8 ± 3.4
CSP						
Onset latency (ms)						
Right	71.7 ± 10.4	71 ± 8.6	73.3 ± 8.7	72.1 ± 13.3	70.9 ± 11.4	72.6 ± 7.8
Left	71.1 ± 11.8	72.5 ± 11.9	71.2 ± 11.7	71.9 ± 13.2	70.3 ± 12.1	71.4 ± 7.8
Duration (ms)						
Right	53.4 ± 16.2	53.9 ± 19	51.4 ± 15.3	53.4 ± 18.8	55.6 ± 15.2	48.8 ± 15.8
Left	57.1 ± 25.0	54.2 ± 8.5	59.8 ± 39.8	50.1 ± 11.8	60.8 ± 23.8 ^§^	50.4 ± 13.1
Offset latency (ms)						
Right	125.2 ± 14.9	124.9 ± 17.8	124.7 ± 13.4	125.5 ± 16	125.6 ± 14.4	121.4 ± 15.5
Left	128.1 ± 22.9	126.7 ± 14.4	130 ± 35.5	122 ± 10.3	131.1 ± 21.5	121.7 ± 12.3
Current intensity (mA)						
Right	57.7 ± 24.6 ^δ^	61.6 ± 25.9 ^δ^	45.1 ± 20.9	62.7 ± 26.8	54.4 ± 24.3 ^§^	30.3 ± 13.4
Left	48.8 ± 21.9 ^δ^	55.1 ± 23.8 ^§^	45 ± 18.6	57.2 ± 26.2 **^§^	49.8 ± 21.8 ^§^	43.7 ± 37.2

CMR: cutaneomuscular reflex; CSP: cutaneous silent period; mA: milliAmpere; ms: milliseconds; * *p* < 0.0001 vs. patients not complaining of dropping objects; ** *p* < 0.05 vs. patients not complaining of dropping objects; ^δ^ *p* < 0.0001 vs. healthy controls; ^§^ *p* < 0.05 vs. healthy controls.

**Table 4:** Summary of quantitative sensory testing results. Log and ln transformed data.

		Carpal Tunnel Syndrome				Healthy Controls
		Dropping Objects			No Dropping Objects	
	All	Small	Large	Large/Small		
CDT						
Dorsum						
Right	1.486 ± 0.033 ^§^**	1.478 ± 0.053	1.480 ± 0.028	1.474 ± 0.032 ^§^**	1.496 ± 0.012	1.500 ± 0.002
Left	1.490 ± 0.025 ^§^	1.481 ± 0.043	1.490 ± 0.026	1.469 ± 0.015	1.495 ± 0.015	1.500 ± 0.002
Index						
Right	1.458 ± 0.101 ^§^	1.415 ± 0.180 ^§^	1.485 ± 0.017	1.464 ± 0.052 ^§^	1.473 ± 0.063	1.492 ± 0.014
Left	1.455 ± 0.168 ^δ^	1.374 ± 0.332 ^§^	1.476 ± 0.042 ^§^	1.483 ± 0.020 ^§^	1.481 ± 0.029	1.495 ± 0.009
Little finger						
Right	1.458 ± 0.059 ^§^**	1.433 ± 0.081	1.435 ± 0.084 ^§^	1.467 ± 0.028	1.477 ± 0.034	1.483 ± 0.019
Left	1.420 ± 0.280 ^§^	1.423 ± 0.084	1.475 ± 0.025	1.467 ± 0.024	1.372 ± 0.437	1.485 ± 0.019
HPT						
Dorsum						
Right	1.636 ± 0.043	1.640 ± 0.036	1.643 ± 0.041	1.661 ± 0.036	1.621 ± 0.047	1.635 ± 0.040
Left	1.633 ± 0.041	1.636± 0.041	1.637 ± 0.039 **	1.649 ± 0.053 **	1.625 ± 0.035	1.638 ± 0.036
Index						
Right	1.663 ± 0.025	1.672 ± 0.021	1.670 ± 0.026	1.664 ± 0.030	1.657 ± 0.026	1.659 ± 0.034
Left	1.638 ± 0.129	1.675 ± 0.012	1.659 ± 0.035	1.656 ± 0.042	1.607 ± 0.189	1.654 ± 0.035
Little finger						
Right	1.658 ± 0.033	1.657 ± 0.035	1.664 ± 0.038	1.680 ± 0.015	1.649 ± 0.031	1.656 ± 0.036
Left	1.657 ± 0.038	1.656 ± 0.40	1.663 ± 0.034	1.667 ± 0.040	1.653 ± 0.030	1.660 ± 0.033
VDT						
Index						
Right	−0.075 ± 1.023 ^δ^	0.232 ± 1.085 ^§^	0.103 ± 1.086 ^§^	−0.134 ± 0.479 ^§^	−0.164 ± 0.998 ^§^	−0.786 ± 0.806
Left	−0.249 ± 1.217 ^δ^	−0.052 ± 1.594 ^§^	−0.192 ± 1.857 ^§^	−0.110 ± 1.432 ^§^	−0.326 ± 1.071 ^§^	−0.987 ± 0.796
Little finger						
Right	−0.315 ± 0.982 ^δ^	−0.187± 0.967 ^§^	−0.351 ± 1.193	0.197 ± 0.600 ^§^	−0.504 ± 1.018	−0.698 ± 0.812
Left	−0.345 ± 0.964 ^δ^	−0.304 ± 1.053 ^§^	−0.231 ± 0.846 ^§^	0.089 ± 0.476 ^§^	−0.429 ± 1.044	−0.869 ± 0.948

CDT: cold detection threshold; HPT: heat-pain threshold; VDT: vibration detection threshold. ** *p* < 0.05 vs. patients not complaining of dropping objects; ^δ^ *p* < 0.0001 vs. healthy controls; ^§^ *p* < 0.05 vs. healthy controls.

## Data Availability

The data sets used and/or analyzed in the current study are available from the first author upon reasonable request.

## Supplementary Materials

The following supporting information can be downloaded at: https://www.mdpi.com/article/10.3390/brainsci13111576/s1, Table S1. Nerve conduction studies, cutanomuscular reflex and cutaneous silent period in patients’ hands with CTS, without CTS and healthy controls’ hands; Table S2: Summary of quantitative sensory testing results. Z-scores; Table S3: Summary of quantitative sensory testing results. Log and ln transformed data in patients’ hands with CTS, without CTS and healthy controls’ hands.

## References

[1] Stevens J.C., Dyck P.J., Thomas P.K. (2005). Median neuropathy. Peripheral Neuropathy.

[2] Padua L., Coraci D., Erra C., Pazzaglia C., Paolasso I., Loreti C., Caliandro P., Hobson-Webb L.D. (2016). Carpal tunnel syndrome: Clinical features, diagnosis, and management. Lancet Neurol..

[3] Pazzaglia C., Caliandro P., Granata G., Tonali P., Padua L. (2010). “Dropping objects”: A potential index of severe carpal tunnel syndrome. Neurol. Sci..

[4] Levine D.W., Simmons B.P., Koris M.J., Daltroy L.H., Hohl G.G., Fossel A.H., Katz J.N. (1993). A self-administered questionnaire for the assessment of severity of symptoms and functional status in carpal tunnel syndrome. J. Bone Jt. Surg. Am..

[5] Zhang W., Johnston J.A., Ross M.A., Sanniec K., Gleason E.A., Dueck A.C., Santello M. (2013). Effects of carpal tunnel syndrome on dexterous manipulation are grip type-dependent. PLoS ONE.

[6] Yen W.J., Kuo Y.L., Kuo L.C., Chen S.M., Kuan T.S., Hsu H.Y. (2014). Precision pinch performance in patients with sensory deficits of the median nerve at the carpal tunnel. Mot. Control.

[7] de la Llave-Rincón A.I., Fernández-de-Las-Peñas C., Pérez-de-Heredia-Torres M., Martínez-Perez A., Valenza M.C., Pareja J.A. (2011). Bilateral deficits in fine motor control and pinch grip force are not associated with electrodiagnostic findings in women with carpal tunnel syndrome. Am. J. Phys. Med. Rehabil..

[8] Tamburin S., Cacciatori C., Marani S., Zanette G. (2008). Pain and motor function in carpal tunnel syndrome. A clinical, neurophysiological and psychophysical study. J. Neurol..

[9] Ortiz-Corredor F., Calambas N., Mendoza-Pulido C., Galeano J., Díaz-Ruíz J., Delgado O. (2011). Factor analysis of carpal tunnel syndrome questionnaire in relation to nerve conduction studies. Clin. Neurophysiol..

[10] Pierrot-Deseillinghy E., Burke D. (2005). Corticomuscular, withdrawal and flexor reflex afferent responses. The Circuitry of the Human Spinal Cord: Its Role in Motor Control and Movement Disorders.

[11] Marchand-Pauvert V., Iglesias C. (2008). Properties of human spinal interneurones: Normal and dystonic control. J. Physiol..

[12] Floeter M.K. (2003). Cutaneous silent periods. Muscle Nerve.

[13] Kofler M., Leis A.A., Valls-Solé J. (2019). Cutaneous silent periods—Part 1: Update on physiological mechanisms. Clin. Neurophysiol..

[14] Caccia M.R., McComas A.J., Upton A.R., Blogg T. (1973). Cutaneous Reflexes in Small Muscles of the Hand. J. Neurol. Neurosurg. Psychiatry.

[15] Serrao M., Parisi L., Pierelli F., Rossi P. (2001). Cutaneous afferents mediating the cutaneous silent period in the upper limbs: Evidence for a role of low-threshold sensory fibers. Clin. Neurophysiol..

[16] Isoardo G., Stella M., Cocito D., Risso D., Migliaretti G., Cauda F., Palmitessa A., Faccani G., Ciaramitaro P. (2012). Neuropathic pain in post-burn hypertrophic scars: A psychophysical and neurophysiological study. Muscle Nerve.

[17] Inghilleri M., Cruccu G., Argenta M., Polidori L., Manfredi M. (1997). Silent period in upper limb muscles after noxious cutaneous stimulation in man. Electroencephalogr. Clin. Neurophysiol..

[18] Aurora S.K., Ahmad B.K., Aurora T.K. (1998). Silent Period Abnormalities in Carpal Tunnel Syndrome. Muscle Nerve.

[19] Svilpauskatie J., Truffert A., Vaiciene N., Magistris M.R. (2006). Cutaneous silent period in carpal tunnel syndrome. Muscle Nerve.

[20] Rolke R., Baron R., Maier C., Tölle T.R., Treede R.D., Beyer A., Binder A., Birbaumer N., Birklein F., Bötefür I.C. (2006). Quantitative sensory testing in the German Research Network on Neuropathic Pain (DFNS): Standardised protocol and reference values. Pain.

[21] Isoardo G., Ciullo S., Titolo P., Fontana E., Battiston B., Stella M., Luxardo N., Laino F., Migliaretti G., Stura I. (2021). The relationship between alexithymia, sensory phenotype and neurophysiological parameters in patients with chronic upper limb neuropathy. J. Neural Transm..

[22] Treede R.D. (2019). The role of quantitative sensory testing in the prediction of chronic pain. Pain.

[23] American Academy of Neurology (1993). Practice parameters for carpal tunnel syndrome. Neurology.

[24] Jablecki C.K., Andary M.T., Floeter M.K., Miller R.G., Quartly C.A., Vennix M.J., Wilson J.R. (2002). Practice parameter: Electrodiagnostic studies in carpal tunnel syndrome. Report of the American Academy of electrodiagnostic Medicine, America Academy of Neurology and the American Academy of Physical Medicine and Rehabilitation. Neurology.

[25] Bouhassira D., Attal N., Alchaar H., Boureau F., Brochet B., Bruxelle J., Cunin G., Fermanian J., Ginies P., Grun-Overdyking A. (2005). Comparison of pain syndromes associated with nervous or somatic lesions and development of a new neuropathic pain diagnostic questionnaire (DN4). Pain.

[26] Jensen M.P., McFarland C.A. (1993). Increasing the Reliability and Validity of Pain Intensity Measurement in Chronic Pain Patients. Pain.

[27] Oxford Grice K., Vogel K.A., Le V., Mitchell A., Muniz S., Vollmer M.A. (2003). Adult Norms for a Commercially Available Nine Hole Peg Test for Finger Dexterity. Am. J. Occup. Ther..

[28] Sobeeh M.G., Ghozy S., Elshazli R.M., Landry M. (2022). Pain mechanisms in carpal tunnel syndrome: A systematic review and meta-analysis of quantitative sensory testing outcomes. Pain.

[29] Zanette G., Cacciatori C., Tamburin S. (2010). Central sensitization in carpal tunnel syndrome with extraterritorial spread of sensory symptoms. Pain.

[30] Enax-Krumova E., Attal N., Bouhassira D., Freynhagen R., Gierthmühlen J., Hansson P., Kuehler B.M., Maier C., Sachau J., Segerdahl M. (2021). Contralateral Sensory and Pain Perception Changes in Patients with Unilateral Neuropathy. Neurology.

[31] Yemisci O.U., Yalbuzdag S.A., Cosar S.N., Oztop P., Karatas M. (2011). Ulnar nerve conduction abnormalities in carpal tunnel syndrome. Muscle Nerve.

[32] Maeda Y., Kettner N., Holden J., Lee J., Kim J., Cina S., Malatesta C., Gerber J., McManus C., Im J. (2014). Functional deficits in carpal tunnel syndrome reflect reorganization of primary somatosensory cortex. Brain.

[33] Castiello U. (2005). The neuroscience of grasping. Nat. Rev. Neurosci..

[34] Muir R.B., Lemon R.N. (1983). Corticospinal neurons with a special role in precision grip. Brain Res..

[35] Garnett R., Stephens J.A. (1981). Changes in the recruitment threshold of motor units produced by cutaneous stimulation in man. J. Physiol..

[36] Kofler M. (2003). Functional organization of exteroceptive inhibition following nociceptive electrical fingertip stimulation in humans. Clin. Neurophysiol..

