# Supplementary material for: Psychophysiological and Neurophysiological Correlates of Dropping Objects from Hands in Carpal Tunnel Syndrome
Source: Brain Sci. 2023 Nov 10;13(11):1576. doi: 10.3390/brainsci13111576 (PMC10670400; doi:10.3390/brainsci13111576)
Supplement: Supplementary file 1 [file brainsci-13-01576-s001.zip › brainsci-2620711-supplementary.pdf]

Supplementary Table S1. Nerve conduction studies, cutanomuscular reflex and cutaneous silent period in patients' hands with CTS, without CTS and healthy controls' hands.

	CTS hands	No-CTS hands	Healthy controls
Median NCS			
SCV (m/s)	40,8 ± 7,0$^{*\delta}$	52.3 ± 3.8 $^{\delta}$	58,5 ± 8.3
SAP-amp (µV)	18.1 ± 13.6$^{*\delta}$	33.5 ± 13.3§	47.8 ± 21.4
MCV (m/s)	52.3 ± 6.1$^{**\delta}$	54.9 ± 4.2§	57.4 ± 4.9
CMAP-amp (mV)	7,2 ± 3.1$^{*\delta}$	8.1 ± 3.8	8.9 ± 4.4
DML (ms)	4.7 ± 1.4$^{*\delta}$	3.6 ± 0.3§	3,3 ± 0.5
Ulnar NCS			
SCV (m/s)	57.0 ± 5.8	56.7 ± 4.8	58.2 ± 6.5
SAP-amp (µV)	30.8 ± 14.8 $^{\delta}$	33,2 ± 18.1§	46.4 ± 21.9
CMR			
Onset latency (ms)	92.2 ± 2.9	92.5 ± 14.9	91.8 ± 16.9
Duration (ms)	22.4 ± 21.4§	19.0 ± 18.9	14.3 ± 15.4
Offset latency (ms)	126,8 ± 21.3	119.8 ± 13.0	119.7 ± 13.8
Current intensity (mA)	15.1 ± 8.2 $^{\delta}$	14.6 ± 8.6 §	8.8 ± 3.6
CSP			
Onset latency (ms)	71.3 ± 11.4	72.3 ± 8.3	72 ± 7.8
Duration (ms)	56.0 ± 21.9§	48.2 ± 10.9	49.5 ± 14.4
Offset latency (ms)	127.3 ± 20.1	121.4 ± 10.0	121.6 ± 14.0
Current intensity (mA)	52.4 ± 23.8$^{\delta}$	48.1 ± 21.8$^{\delta}$	40.9 ± 27.4

CMAP-amp: compound muscle action potential amplitude; CMR: cutaneomuscular reflex; CSP: cutaneous silent period; CTS: carpal tunnel syundrome; DML: distal motor latency; m: meters; MCV: motor conduction velocity; mA: milliAmpere; ms: milliseconds; mV: milliVolt; NCS: nerve conduction studies; s: seconds; SAP-amp: sensory action potential amplitude; SCV: sensory conduction velocity; µV: micronVolt. * p<0.0001 vs patients' hands without carpal tunnel syndrome. ** p<0.05 vs patients' hands without carpal tunnel syndrome. $^{\delta}$ p<0.0001 vs healthy controls' hands. § p <0.05 vs healthy controls' hands.

Supplementary Table S2. Summary of quantitative sensory testing results. Z-scores.

	Carpal tunnel syndrome				
		Dropping objects			No dropping objects
	All	Small	Large	Large/small	
CDT					
Dorsum					
Right	- 8.24 ± 15.5	- 8.04 ± 20.88	- 7.26 ± 10.1	- 9.28 ± 11.7	- 1.31 ± 4.52
Left	- 5.50 ± 11.8	- 7.5 ± 16.3	- 3,91 ± 10.14	- 4.28 ± 6.02	- 2.05 ± 5.88
Index					
Right	- 3.34 ± 9.20	- 5.74 ± 13.3	- 0.62 ± 1.30	- 2.1 ± 3.89	- 1.52 ± 4.65
Left	- 6.67 ± 24.1	- 13.4 ± 36.4	- 2.14 ± 4.70	- 1.35 ± 2.24	- 1.56 ± 3.18
Little finger					
Right	- 2.15 ± 3.79**	- 2.71 ± 4.34**	- 2.55 ± 4.49	- 0.81 ± 1.53	- 0.40 ± 1.88
Left	- 1.94 ± 3.27	- 3.45 ± 4.52	- 0.62 ± 1.39	- 1.9 ± 1.31	-6.17 ± 23.5
HPT					
Dorsum					
Right	0.29 ± 0.94**	0.14 ± 0.91	0.2 ± 1.03	0.66 ± 0.89**	- 0.32 ± 1.17
Left	0.09 ± 1.23	- 0.02 ± 1.16	- 0.01 ± 1.11	0.33 ± 1.5	- 0.32 ± 1
Index					
Right	0.38 ± 0.71**	0.44 ± 0.62	0.39 ± 0.77	0.23 ± 0.88**	0.03 ± 0.78
Left	0.4 ± 0.88**	0.70 ± 0.34	0.22 ± 1.01	0.14 ± 1.2	- 1.26 ± 5.44
Little finger					
Right	0.23 ± 0.94	0.04 ± 0.99	0.22 ± 1.07	0.67 ± 0.42**	- 0.19 ± 0.88
Left	0.08 ± 1.07	- 0.03 ± 1.15	0.11 ± 0.39	0.03 ± 1.16	- 0.18 ± 0.87
VDT					
Index					
Right	1.20 ± 1.17	1.35 ± 1.33	1.19 ± 1.33	0.9 ± 0.58	0.86 ± 1.22
Left	1.25 ± 1.80	1.33 ± 2.18	1.14 ± 1.14	1.25 ± 1.96	0.95 ± 1.46
Little finger					
Right	1.93 ± 1.15	0,87 ± 1.17	0.65 ± 1.45	1.32 ± 0.78	0.47 ± 1.23
Left	0.78 ± 1.04	0.69 ± 1.31	1.77 ± 0.96	1.94 ± 0.54	0.55 ± 1.18

CDT: cold detection threshold; HPT: heat-pain threshold; VDT: vibration detection threshold. * p<0.0001 vs patients not complaining of dropping objects. ** p< 0.05 vs patients not complaining of dropping objects.

Supplementary Table S3. Summary of quantitative sensory testing results. Log and ln transformed data in patients' hands with CTS, without CTS and healthy controls' hands.

	CTS hands	No-CTS hands	Healthy controls
CDT			
Dorsum	1.489 ± 0.03	1.487 ± 0.02	1.500 ±0.002
Index	1.450 ± 0.15§	1.482 ± 0.03	1.494 ± 0.010
Little finger	1.457 ± 0.002§	1.470 ± 0.42	1.484 ± 0.02
HPT			
Dorsum	1.638 ± 0.05	1.620 ± 0.030	1.638 ± 0.04
Index	1.658 ± 0.10	1.646 ± 0.003	1.657 ± 0.03
Little finger	1.660 ± 0.03	1.649 ± 0.04	1.658 ± 0.03
VDT			
Index	- 0.113 ± 1.09 δ	- 0.32 ± 1.19§	- 0.88 ± 0.80
Little finger	- 0.287 ± 0.92 §	- 0.48 ± 1.11	- 0.78 ± 0.87

CDT: cold detection threshold; HPT: heat-pain threshold; VDT: vibration detection threshold. * $p<0.0001$ vs patients' hands without carpal tunnel syndrome. ** $p<0.05$ vs patients' hands without carpal tunnel syndrome. δ $p<0.0001$ vs healthy controls' hands. § $p <0.05$ vs healthy controls' hands.
